# Humoral and Cellular Immune Responses to SARS-CoV-2 mRNA Vaccination in Patients with Multiple Sclerosis: An Israeli Multi-Center Experience Following 3 Vaccine Doses

**DOI:** 10.3389/fimmu.2022.868915

**Published:** 2022-04-01

**Authors:** Ron Milo, Elsebeth Staun-Ram, Dimitrios Karussis, Arnon Karni, Mark A. Hellmann, Erez Bar-Haim, Ariel Miller, The Israeli Neuroimmunology Study Group on COVID-19 Vaccination in Multiple Sclerosis, Lea Glass-Marmor

**Affiliations:** ^1^Department of Neurology, Barzilai Medical Center, Ashkelon & Faculty of Health Sciences, Ben-Gurion University of the Negev, Beer-Sheva, Israel; ^2^Multiple Sclerosis Center and Neuroimmunology Unit, Carmel Medical Center & Rappaport Faculty of Medicine, Technion-Israel Institute of Technology, Haifa, Israel; ^3^Unit for Neuroimmunology, Multiple Sclerosis and Cell Therapy, Hadassah Medical Center & Faculty of Medicine, Hebrew University, Jerusalem, Israel; ^4^Neuroimmunology and Multiple Sclerosis Unit, Tel Aviv Sourasky Medical Center & Sackler Faculty of Medicine, Sagol School of Neuroscience, Tel Aviv University, Tel Aviv, Israel; ^5^Department of Neurology, Rabin Medical Center & Sackler Faculty of Medicine, Tel Aviv University, Tel Aviv, Israel; ^6^Department of Biochemistry and Molecular Genetics, Israel Institute for Biological Research, Ness Ziona, Israel

**Keywords:** autoimmunity, COVID-19, humoral response, IgG, multiple sclerosis, SARS-CoV-2, T-cell immune response, disease modifying therapies (DMTs)

## Abstract

**Background:**

Immunomodulatory/immunosuppressive activity of multiple sclerosis (MS) disease modifying therapies (DMTs) might affect immune responses to SARS-CoV-2 exposure or vaccination in patients with MS (PwMS). We evaluated the effect of DMTs on humoral and cell-mediated immune responses to 2 and 3 vaccinations and the longevity of SARS-Cov-2 IgG levels in PwMS.

**Methods:**

522 PwMS and 68 healthy controls vaccinated with BNT162b2-Pfizer mRNA vaccine against SARS-CoV-2, or recovering from COVID-19, were recruited in a nation-wide multi-center study. Blood was collected at 3 time-points: 2-16 weeks and ~6 months post 2^nd^ vaccination and 1-16 weeks following 3^rd^ vaccination. Serological responses were measured by quantifying IgG levels against the spike-receptor-binding-domain of SARS-CoV-2, and cellular responses (in a subgroup analysis) by quantifying IFNγ secretion in blood incubated with COVID-19 spike-antigen.

**Results:**

75% PwMS were seropositive post 2^nd^ or 3^rd^ vaccination. IgG levels decreased by 82% within 6 months from vaccination (p<0.0001), but were boosted 10.3 fold by the 3^rd^ vaccination (p<0.0001), and 1.8 fold compared to ≤3m post 2^nd^ vaccination (p=0.025). Patients treated with most DMTs were seropositive post 2^nd^ and 3^rd^ vaccinations, however only 38% and 44% of ocrelizumab-treated patients and 54% and 46% of fingolimod-treated patients, respectively, were seropositive. Similarly, in COVID-19-recovered patients only 54% of ocrelizumab-treated, 75% of fingolimod-treated and 67% of cladribine-treated patients were seropositive. A time interval of ≥5 months between ocrelizumab infusion and vaccination was associated with higher IgG levels (p=0.039 post-2^nd^ vaccination; p=0.036 post-3^rd^ vaccination), and with higher proportions of seropositive patients. Most fingolimod- and ocrelizumab-treated patients responded similarly to 2^nd^ and 3^rd^ vaccination. IFNγ-T-cell responses were detected in 89% and 63% of PwMS post 2^nd^ and 3^rd^ vaccination, however in only 25% and 0% of fingolimod-treated patients, while in 100% and 86% of ocrelizumab-treated patients, respectively.

**Conclusion:**

PwMS treated with most DMTs developed humoral and T-cell responses following 2 and 3 mRNA SARS-CoV-2 vaccinations. Fingolimod- or ocrelizumab-treated patients had diminished humoral responses, and fingolimod compromised the cellular responses, with no improvement after a 3^rd^ booster. Vaccination following >5 months since ocrelizumab infusion was associated with better sero-positivity. These findings may contribute to the development of treatment-stratified vaccination guidelines for PwMS.

## Introduction

With the outbreak of the COVID-19 pandemic and the rapid development of SARS-CoV-2 vaccines worldwide, concern was raised that the immunomodulatory/immunosuppressive effects of multiple sclerosis (MS) disease modifying therapies (DMTs) might affect COVID-19 disease severity as well as the development of humoral and cellular immunity after SARS-CoV-2 exposure or vaccination (1). In Israel, a nationwide COVID-19 vaccination program was rapidly launched starting December 2020, based solely upon the BNT162b2 (Pfizer-BioNTech) mRNA vaccine, offering two doses administered 3 weeks apart, and a third vaccine booster ≥ 5 months after the 2^nd^ dose (2), a vaccination regime associated with a good safety profile (3). All PwMS are recommended by the National Multiple Sclerosis Society of the United States and by the MS International Federation to be vaccinated against SARS-CoV-2 (4, 5). With the variety of available DMTs, that exert their action through diverse immunological mechanisms, it is of high importance to investigate the immune response to SARS-CoV-2 vaccination in PwMS treated with various immunotherapies, in order to develop optimal and treatment-stratified guidelines. The immune response to SARS-CoV-2 consists of both a cellular and a humoral response, but measurement of SARS-CoV-2 IgG is widely used to identify persons who have recovered from COVID-19 infection or as a confirmation of a sufficient vaccine response, based on the fact that the levels of neutralizing antibodies have been shown to be highly predictive of SARS-CoV-2 immune protection (6). Initial reports have demonstrated that while most MS patients show a positive humoral immune response shortly after the 2^nd^ SARS-CoV-2 mRNA vaccination, a considerable proportion of patients treated with fingolimod or ocrelizumab do not develop antibodies (7–12). In light of the immunological effects of the DMTs, it is important to evaluate the effect of the patients’ immune response to the vaccine and to COVID-19 over time and the effects of the third dose of vaccine in patients treated with ocrelizumab and fingolimod. In this nation-wide multi-center prospective study we examined the levels of anti-SARS-CoV-2 antibodies in PwMS treated with most available DMTs and in healthy controls (HC), during a period of at least 6 months following the 2^nd^ SARS-CoV-2 vaccination and at 1-16 weeks following a 3^rd^ vaccine booster. In addition, we evaluated the cell-mediated immune responses against the COVID-19 spike protein in a subgroup of the participants.

## Materials and Methods

### Participants and Sampling

This observational multi-center prospective study was conducted in the MS centers at 5 major Israeli hospitals: Hadassah Medical Center, Tel Aviv Sourasky Medical Center, Barzilai Medical Center, Rabin Medical Center and Carmel Medical Center, in compliance with the principles of the Declaration of Helsinki, as approved by the Ethics Committees of each hospital. The study included 522 PwMS and 68 HC, that were recently vaccinated or about to be vaccinated against SARS-CoV-2 with the mRNA vaccine BNT162b2 (Pfizer-BioNTech), or who recovered from COVID-19. All participants signed a written informed consent prior to the study procedures. Demographic and clinical data were recorded at recruitment. Serum was collected 2-16 weeks (mean=7.5 weeks, median=6.9 weeks) after the 2^nd^ vaccine dose (time-point 1), ~6 months (17-39 weeks, mean and median=25 weeks.) after the 2^nd^ vaccine dose (time-point 2) and 1-16 weeks (mean=7.2 weeks, median=6.7 weeks) after the 3^rd^ vaccine booster (time-point 3) and kept at -80^0^C until assessed. In a subgroup of 39 PwMS and 8 HC, whole blood was collected at the same time-points and used for assessment of the anti-spike protein T-cell response.

### SARS-CoV-2 IgG

The humoral response was measured at a centralized laboratory (the Clinical Virology Laboratory at Hadassah Medical Center, Jerusalem) using the spike receptor-binding domain (RBD) Architect SARS-CoV-2 IgG II Quant Assay (Abbott). Serum positivity was determined in accordance with the definition of The Israeli Health Ministry at: ≥50 arbitrary units per ml in vaccinated and/or COVID-19-recovered persons.

### T Cell Reactivity

For evaluation of SARS-CoV-2 spike-specific T-cell responses, a quantitative assay SARS-CoV-2 IGRA stimulation tube set (EuroImmun, Germany) was used according to manufacturer’s protocol. The IFNγ-T cell response assay was carried out at the Israel Institute for Biological Research (Ness Ziona, Israel). Heparinized whole-blood samples were stimulated *ex-vivo* with the COVID-19 spike protein for 24 hours, then plasma was collected and secreted IFNγ was quantified by ELISA (ELISA DuoSet, R&D Systems, Minneapolis, Minnesota, USA). A similar sample without antigen stimulation was used as control. Values above 25 pg/ml of IFNγ spike-specific response were considered positive.

### Statistical Analysis

Data was analyzed using SPSS v24. The antibody levels were transformed on a Log10 scale, to normalize their distribution. A general univariate linear model with Bonferonni adjustment for multiple comparisons was used to compare IgG levels between groups of demographic or clinical variables, adjusted for time from vaccination/infection to blood collection (as samples were collected within a relatively large interval of 2-16 weeks post-vaccine or longer post-COVID-19), age and gender. Since the number of samples from each participant varied, we used a generalized linear model (generalized estimated equation model) to compare IgG levels between time-points, where comparison of 1^st^ and 3^rd^ time-point was adjusted for time from vaccination to blood collection. A multiple linear regression model was used to assess correlation between IgG levels and variables, adjusted for time between vaccination and blood collection, age or gender. P value at <0.05 was considered significant.

## Results

### Demographic and Clinical Characteristics of the Participants

We recruited for this prospective study 522 PwMS and 68 HC, who were vaccinated, about to be vaccinated, or who had been infected with SARS-CoV-2. **Table 1** summarizes their demographic and clinical characteristics. There was no significant difference between PwMS and HC regarding gender, BMI and smoking status, with the exception of a small age difference between the healthy participants and the MS patients (50 vs. 46 years, p=0.013). 93% of PwMS and 96% of HC received the COVID-19 vaccine, and 61 PwMS (12%) and 5 HC (7.4%) had a confirmed COVID-19 infection before (44 and 4, respectively) or during the study (17 and 1, respectively). COVID-19 infection was reported in 10 PwMS following the 2^nd^ vaccination and in 3 patients following the 3^rd^ vaccine booster, while in none of the HC after these vaccinations.

**Table 1:** Demographic and clinical characteristics of study participants.

Study Population	MS	Healthy	p-value
N	522	68	
Age (years)	46 ± 0.6	50 ± 1.7	**0.013**
Gender Female N (%)	336 (65%)	36 (53%)	0.4
Smoking (Yes)	16.6%	12.3%	0.5
BMI mean ± SE (N)	24 ± 0.3 (219)	25 ± 0.6 (32)	0.3
EDSS (N)	3.2 ± 0.1 (480)		
Vaccinated N (%):	484 (93%)	65 (95.5%)	
No vaccines and no COVID-19	11	0	
1 vaccine only + COVID-19	10	0	
≥2 vaccines	474	65	
3 vaccines	186	23	
Confirmed COVID-19 N (%):	61 (12.0)	5 (7.4)	
only COVID-19, no vaccine	26	3	
Pre- vaccination	18	1	
Post 1^st^ vaccine	4	1	
Post 2^nd^ vaccine	10	0	
Post 3^rd^ vaccine	3	0	
MS Therapy (N):			
Untreated	47		
Interferon beta	33		
Glatiramer Acetate	33		
Natalizumab	38		
S1PR modulator	63		
Teriflunomide	21		
Alemtuzumab	6		
Cladribine	35		
Dimethyl/Diroximel Fumarate	81		
Anti CD-20 monoclonal antibody	155		
Azathioprine	3		
other	6		

F, Female; M, Male; DMT, Disease modifying therapy; EDSS, Expanded Disability Status Scale; S1PR, Sphingosine 1-phosphate receptor.Bold, significant p-value.

### Serological Response in Vaccinated and Post-COVID-19 Participants

**Figure 1:**
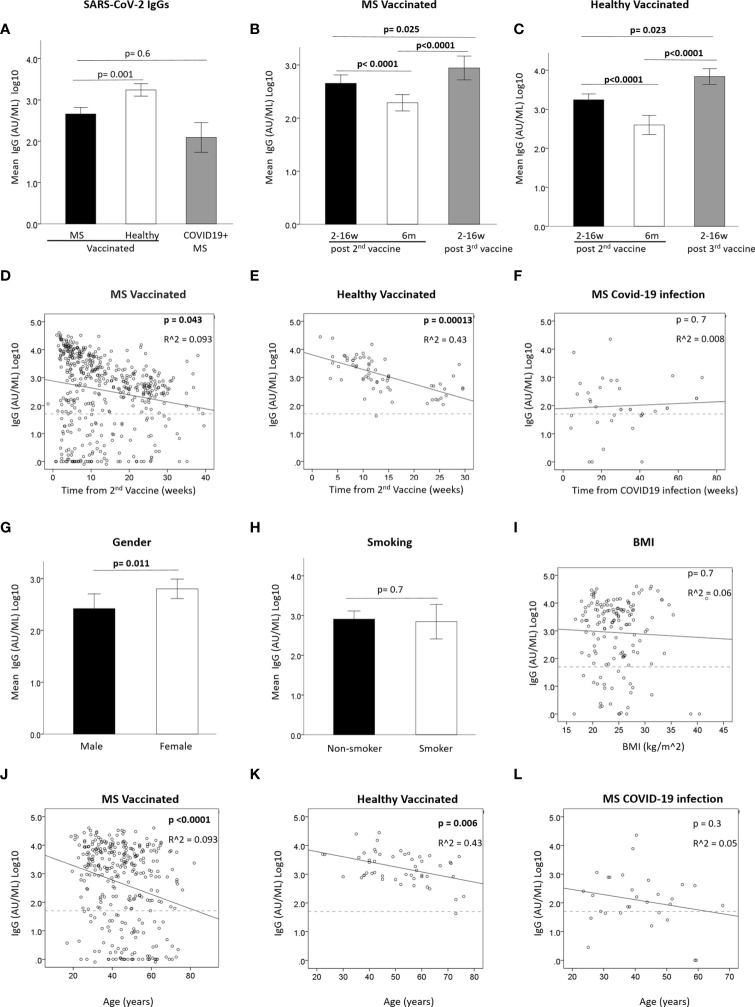
**(A)** Mean IgG levels after 2^nd^ vaccine in vaccinated PwMS (N=309) and healthy (N=48) and in unvaccinated COVID-19-recovered PwMS (N=29). **(B, C)** Mean IgG level in vaccinated PwMS (N=479) **(B)** or healthy (N=65) **(C)** at 3 time-points: <3months and 6 months from 2^nd^ vaccine and <3 months from 3^rd^ vaccine. **(D, E)** Correlation between IgG and time since 2^nd^ vaccine in vaccinated PwMS (N=311) **(D)** or healthy) (N=46) **(E)**. **(F)** Correlation between IgG and time since COVID-19 infection in unvaccinated PwMS. **(G)** mean IgG difference between male (N=107) and female (N=204) PwMS after 2^nd^ vaccine. **(H)** mean IgG difference between non-smokers (N=166) and smokers (N=37) PwMS after 2^nd^ vaccine. **(I)** Correlation between IgG post 2^nd^ vaccine and BMI in PwMS (N=144). **(J, K)** Correlation between IgG after 2^nd^ vaccine and age in vaccinated PwMS (N=311) **(J)** or healthy (N=46) **(K)**. **(L)** Correlation between IgG and age in unvaccinated PwMS recovered from COVID-19 (N=38). Dashed horizontal grey line represents minimum seropositive border (log10(50AU/ml).

The serological response to SARS-CoV-2 vaccine was measured by detecting the anti-SARS-CoV-2 IgG level at 3 time-points: 2-16 weeks (≤3 months) and at 17-39 weeks (~6 months) after the 2^nd^ vaccination, and at 1-16 weeks (≤3 months) after the 3^rd^ vaccine booster. **Table 2** summarizes the IgG levels at each time-point in vaccinated PwMS and HC, as well as the IgG levels post-COVID-19 infection in unvaccinated PwMS. Supplementary data on additional DMTs can be found in **Supplementary Table 1**. 322 samples were available at ≤3 months from 2^nd^ vaccination (mean=7.5 weeks, median=6.9 weeks), 159 samples at ~6 months from 2^nd^ vaccination (mean=25.1 weeks, median=24.6 weeks) and 172 samples at ≤3 months following the 3^rd^ vaccine booster in PwMS (mean=7.2 weeks, median=6.7 weeks). In total, 75.5% of PwMS and 98% of HC were seropositive after the 2^nd^ vaccination, and 78.5% PwMS and 100% HC were seropositive after the 3^rd^ vaccine booster. After 2 vaccinations, the mean IgG level was slightly lower in PwMS compared to HC (p=0.001) (**Figure 1A**). 72% of COVID-19-recovered and non-vaccinated patients had positive IgG levels, obtained after an average time of 30 weeks (median=27 weeks) post- infection, not different from the mean IgG level in vaccinated PwMS (p=0.6), when adjusted for time since infection/vaccination (**Table 1** and **Figure 1A**). IgG levels dropped by 82% in MS patients [OR=0.53, 95%CI: (0.47, 0.59), p<0.0001] and by 76% in healthy individuals [OR=0.50, 95%CI: (0.40, 0.63), p<0.0001] at time-point 2, 6 months after the 2^nd^ vaccination (**Figures 1B, C**). In PwMS the 3^rd^ vaccine booster increased the IgG levels at time-point 3 10 fold compared with the levels at time-point 2 (~6 months post 2^nd^ vaccine) [OR=2.19, 95%CI: (1.75, 2.73), p<0.0001], and 1.8 fold compared with the levels at time-point 1 (≤3 months post 2^nd^ vaccine) [OR=1.21, 95%CI: (1.02, 1.43), p=0.025] (**Figure 1B**). In the healthy participants the 3^rd^ vaccine booster similarly increased the IgG levels 12.6 fold compared with the levels at time-point 2 [OR=3.58, 95%CI: (2.76, 4.64), p<0.0001], and 3 fold compared with the levels at time-point 1 [OR=1.40, 95%CI: (1.05, 1.87), p=0.023] (**Figure 1C**). While IgG levels correlated negatively with time since the 2^nd^ vaccination in vaccinated MS patients [B=-0.038 ± 0.019, 95%CI: (-0.075, -0.001), p=0.043] (**Figure 1D**) and in vaccinated healthy individuals [B=-0.08 ± 0.019, 95%CI: (-0.12, -0.04), p=0.00013] (**Figure 1E**), no correlation was found between IgG levels and time since COVID-19 infection (COVID-19- recovered, unvaccinated patients), measured for a time interval of 4-72 weeks (mean=30 weeks) [B=0.005 ± 0.012, 95%CI: (-0.02, 0.03),p=0.7] (**Figure 1F**).

**Table 2:** IgG index following SARS-CoV-2 vaccination or COVID-19.

IgG AU/mL Mean ± SE [range] (% IgG positive)	Post COVID-19 no vaccine	Time-point 1: ≤3m Post 2^nd^ vaccine	Time-point 2: ~6m Post 2^nd^ vaccine	Time-point 3: ≤3m Post 3^rd^ vaccine
**MS patients**				
**Untreated**	N=2	N=28	N=17	N=14
	661 ± 480	7778 ± 1502	1269 ± 681	18640 ± 4167
	[180 – 1141]	[770 – 32497]	[120 – 12046]	[454 – 40000]
	(100%)	(100%)	(100%)	(100%)
**Alemtuzumab**	N=2	N=3	N=1	
	73 ± 0	8414 ± 3888	1366	
	[73−73]	[761 – 13329]	(100%)	
	(100%)	(100%)		
**Anti CD-20 mAb (Ocrelizumab)**	N=13	N=89	N=38	N=43
	709 ± 586	290 ± 113	137 ± 37	2072 ± 938
	[0 – 7722]	[0 − 7722]	[0 – 992]	[0 – 36108]
	(54%)	(38%)	(42%)	(44%)
**Anti CD-20 mAb (Ofatumumab)**		N=4		N=2
		2120 ± 1344		5210 ± 5021
		[120 – 5826]		[189−10230]
		(100%)		(100%)
**Cladribine**	N=3	N=23	N=11	N=9
	13661 ± 13172	5923 ± 1096	806 ± 181	9826 ± 2691
	[44 – 40000]	[162 – 22148]	[42 – 1991]	[643 – 23144]
	(67%)	(100%)	(91%)	(100%)
**Dimethyl Fumarate**	N=2	N=52	N=28	N=32
	181 ± 75	8401 ± 1217	708 ± 139	13813 ± 2226
	[106 – 256]	[610 – 32938]	[6 – 3813]	[420 – 40000]
	(100%)	(100%)	(96%)	(100%)
**Glatiramer Acetate**		N=22	N=9	N=8
		6984 ± 1577	1283 ± 633	10748 ± 2567
		[153 – 33131]	[152 – 6146]	[2902 – 21115]
		(100%)	(100%)	(100%)
**Interferon β (Avonex, Plegridy, Betaferon, Rebif)**	N=1	N=19	N=15	N=13
	22699	7832 ± 2182	1649 ± 971	16509 ± 3639
	(100%)	[248 – 40000]	[35 – 14783]	[1649 – 40000]
		(100%)	(93%)	(100%)
**Natalizumab**	N=3	N=19	N=12	N=14
	288 ± 164	9698 ± 2458	1372 ± 573	7149 ± 2592
	[90 – 614]	[161 – 40000]	[259 – 7258]	[792 – 33990]
	(100%)	(100%)	(100%)	(100%)
**S1PR modulator (Fingolimod)**	N=4	N=37	N=13	N=24
	298 ± 164	1494 ± 670	200 ± 143	325 ± 191
	[29 – 777]	[0 – 19543]	[0 – 1856]	[0 – 4586]
	(75%)	(54%)	(31%)	(46%)
**S1PR modulator (Siponimod)**	N=1	N=3	N=1	
	25	1278 ± 1095	52	
	(0%)	[69−3464]	(100%)	
		(100%)		
**Teriflunomide**		N=15	N=7	N=10
		6054 ± 1356	2063 ± 1240	13886 ± 4039
		[439 – 16216]	[244 – 9366]	[568 – 40000]
		(100%)	(100%)	(100%)
**Healthy**		N=53	N=16	N=21
		3537 ± 725	855 ± 371	10765 ± 2096
		[42 – 27876]	[108 – 5790]	[787 – 33761]
		(98%)	(100%)	(100%)

AU, arbitrary units; IgG, immunoglobulin G; m, months; mAb, monoclonal antibody; SE, standard error; S1P, Sphingosine 1-phosphate receptor.

### Effect of Demographic Factors on the Serological Response

We assessed whether the serological response after the 2^nd^ vaccination was affected by demographic parameters (**Figures 1G–L**). Mean IgG levels were higher in female than in male PwMS (F=6.49, p=0.011) (**Figure 1G**). No gender-related difference was found in the small cohort of HC (data not shown). No difference was found in PwMS between smokers and non-smokers (F=0.1, P=0.7) (**Figure 1H**), and IgG levels did not correlate with body mass index (BMI) [B=-0.009 ± 0.024, 95%CI: (-0.056, -0.038), p=0.7] (**Figure 1I**). Age negatively correlated with IgG levels, both in vaccinated MS patients [B= -0.026 ± 0.006, 95%CI: (-0.038,-0.015), p<0.0001] (**Figure 1J**) and in vaccinated healthy individuals [B=-0.015 ± 0.005, 95%CI: (-0.025, -0.004), p=0.006] (**Figure 1K**), but not in MS patients post-COVID-19 infection [B=-0.018± 0.016, 95%CI: (-0.05, 0.014), p=0.3] (**Figure 1L**).

### Effect of DMTs on the Serological Response

**Table 2** and **Supplementary Table 1** present the IgG levels according to the DMT that patients were receiving. Patients treated with most DMTs had a positive IgG response both after the 2^nd^ and the 3^rd^ vaccination, with IgG levels similar to untreated MS patients. However patients treated with the S1PR1 modulator fingolimod and the anti-CD20 therapy ocrelizumab had lower IgG levels after both the 2^nd^ and 3^rd^ vaccination (both p<0.0001), with only 38% of ocrelizumab-treated patients and 54% of fingolimod-treated patients being seropositive after the 2^nd^ vaccine dose, and 44% and 46% respectively being seropositive after the 3^rd^ vaccine booster (**Table 2**). Patients receiving other S1PR-modulators ponesimod or siponimod were seropositive both after the 2^nd^ and the 3^rd^ vaccination, but with relatively low IgG levels compared to untreated patients. Interestingly, patients receiving the anti-CD-20 therapy ofatumumab were all seropositive both after the 2^nd^ and the 3^rd^ vaccination, with relatively normal IgG levels (**Table 2** and **Supplementary Table 1**). In contrast, two patients receiving rituximab (a first generation anti-CD20 therapy) had very low IgG levels, only one being borderline seropositive (**Supplementary Table 1**). 100% of cladribine-treated patients were seropositive after the 2^nd^ and the 3^rd^ vaccination, and their IgG levels were comparable to untreated patients (**Table 2**). Although the number of unvaccinated patients who recovered from COVID-19 infection was small (N=32), a similar trend was observed with a positive IgG response in patients under treatment with most DMTs, with the exception of ocrelizumab [7/13 (54%) positivity], fingolimod [3/4 (75%) positivity], siponimod (0/1 positivity) and cladribine [2/3 (67%) positivity] at time of sample collection (**Table 2**).

### Relation Between Time Since Last Ocrelizumab Infusion and IgG Response

**Figure 2:**
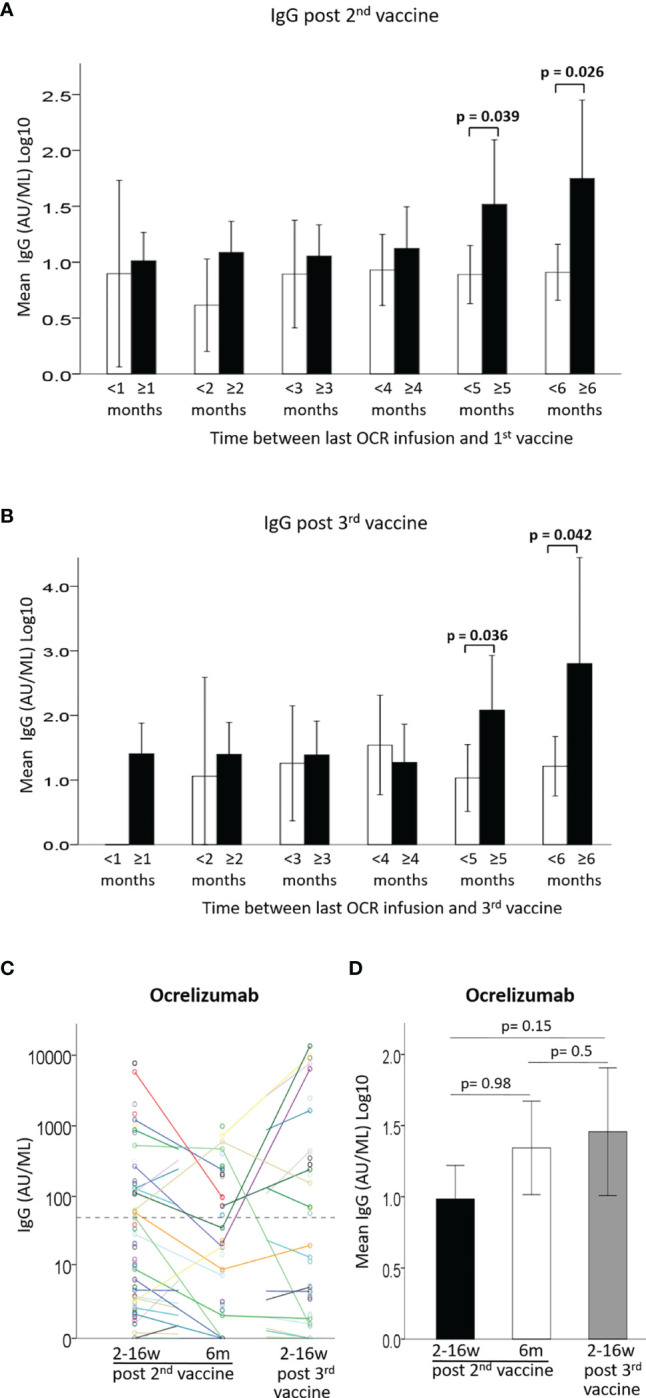
**(A)** Mean IgG levels after 2^nd^ vaccine in patients with < (white) or ≥ (black) 1-6 months between last Ocrelizumab infusion and 1^st^ vaccine. **(B)** Mean IgG levels after 3^rd^ vaccine in patients with < (white) or ≥ (black) 1-6 months between last Ocrelizumab infusion and 3^rd^ vaccine. **(C)** IgG levels of Ocrelizumab-treated patients at 3 time-points: ≤3months and 6 months from 2^nd^ vaccine and ≤3 months from 3^rd^ vaccine. **(D)** Comparison of IgG levels in Ocrelizumab-treated patients at 3 time-points: ≤3months (N=89) and 6 months (N=38) from 2^nd^ vaccine and ≤3 months from 3^rd^ vaccine (N=43). Dashed horizontal grey line represents minimum seropositive border (log10(50AU/ml).

**Figure 3:**
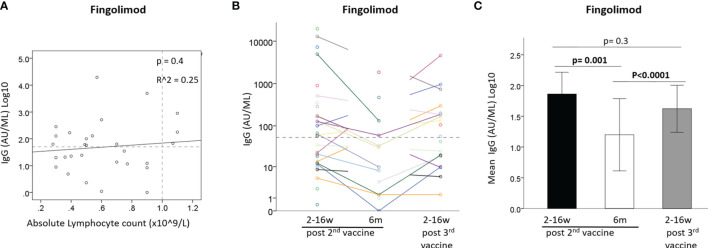
**(A)** Correlation between IgG after 2^nd^ vaccine and absolute lymphocyte count in Fingolimod-treated patients (N=29). **(B)** IgG levels of Fingolimod-treated patients at 3 time-points: ≤3months and 6 months from 2^nd^ vaccine and ≤3 months from 3^rd^ vaccine. **(C)** Mean IgG levels in Fingolimod-treated patients at 3 time-points: ≤3months and 6 months from 2^nd^ vaccine and ≤3 months from 3^rd^ vaccine. Dashed horizontal grey line represents minimum seropositive border (log10(50AU/ml), dashed vertical line represent normal lymphocyte count border.

Since anti-CD20 therapies are expected to have a lowering effect on antibody production after vaccination, and since ocrelizumab is administered periodically every 6 months, it is important to appreciate the preferred timing for immunization between treatment doses. We evaluated the anti SARS-CoV-2 spike protein IgG levels according to the time interval between last ocrelizumab infusion and vaccine administration (**Figure 2**, **Supplementary Figure 1AB** and **Supplementary Table 2**). Only a very weak, although significant, correlation was found between the time since previous ocrelizumab infusion and IgG levels after the 2^nd^ vaccination [B=0.02 ± 0.01, 95%CI: (0.0, 0.04), p=0.048] (**Supplementary Figure 1A**) or after the 3^rd^ vaccine booster [B=0.048 ± 0.022, 95% CI (0.00, 0.09), p=0.041] (**Supplementary Figure 1B**). Mean IgG levels were significantly higher in patients with a ≥ 5 months interval between last infusion and the 1^st^ vaccine dose (**Figure 2A** and **Supplementary Table 2**) or the 3^rd^ vaccine booster (**Figure 2B** and **Supplementary Table 2**), compared to patients with an interval of < 5 months (F=4.43, p=0.039 and F=4.76, p=0.036, respectively). Similarly, patients with a ≥6 months interval had significantly higher IgG levels than patients with < 6 months between last infusion and 1^st^ (**Figure 2A**) or 3^rd^ vaccine (**Figure 2B**) (F= 5.19, p=0.026 and F=4.43, p=0.042, respectively) (**Supplementary Table 2**). Furthermore, the percentage of seropositive patients was higher in patients with ≥5 months interval than in those with <5 months interval between last infusion and 1^st^ or 3^rd^ vaccine (44% vs. 26% and 62% vs. 30%, respectively), and higher in patients with ≥6 months interval than in those with <6 months interval (60% vs. 25% and 75% vs. 36%, respectively) **(** **Supplementary Table 2**). Comparing the IgG levels in ocrelizumab-treated patients after the 2^nd^ and 3^rd^ vaccinations revealed a similar pattern for most patients, e.g. those who had a positive response after the first 2 vaccine doses were most likely to be also seropositive after the 3^rd^ vaccination, while most patients with an insufficient response after the 2^nd^ vaccination remained mostly seronegative after the 3^rd^ vaccination, although some patients did benefit from the booster (**Figure 2C**). Using a generalized linear model to compare IgG levels between the 3 time-points we found no differences between time-point 1 and 2 post 2^nd^ vaccination [OR1.00, 95%CI: (0.73, 1.39), p=0.98], between time-point 2 post 2^nd^ vaccine and time-point 3 post 3^rd^ vaccination [OR1.25, 95%CI: (0.70, 2.2), p=0.5], or between time-point 1 post-2^nd^ vaccination and time-point 3 post-3^rd^ vaccination [OR1.38, 95%CI: (0.89, 2.1), p=0.2] (**Figure 2D**).

### IgG Levels in Fingolimod-Treated Patients

IgG levels did not correlate with absolute lymphocyte counts at time of vaccination in fingolimod-treated patients [B=0.537 ± 0.64, 95%CI: (-0.77, 1.85), p=0.4] (**Figure 3A**). Comparison of the IgG response after the 2^nd^ and 3^rd^ vaccination showed a similar pattern for most fingolimod-treated patients, with seronegative patients remaining negative also after the 3^rd^ vaccination (**Figure 3B**). A generalized linear model comparing IgG levels between the 3 time-points revealed that the IgG levels were reduced between time-point 1 and 2 after the 2^nd^ vaccination [OR=0.53, 95%CI: (0.36, 0.78), p=0.001], and increased between 2^nd^ and 3^rd^ time-point following the vaccine booster [OR=1.88, 95%CI: (1.42, 2.49), p<0.0001]; however, the 3^rd^ vaccine booster did not increase the IgG levels further than at time-point 1 (post 2^nd^ vaccination) [OR=0.76, 95%CI: (0.48, 1.21), p=0.3] (**Figure 3C**).

### INFγ Immune Cell Response

T-cell immune response to the vaccine was assessed by measuring IFNγ secretion in response to incubation of whole blood with the SARS-CoV-2 spike protein. Data were available from 26 patients and 6 healthy participants after 2^nd^ vaccine, along with their serological assay, and from 16 patients and 2 healthy participants after the 3^rd^ vaccine booster (**Table 3** and **Supplementary Table 3**). There was no significant difference in IFNγ-T-cell response levels between PwMS and healthy participants after the 2^nd^ vaccination (p=0.8) or after the 3^rd^ vaccine booster (p=0.2). 100% of healthy participants and 88.5% of MS patients had a positive IFNγ-T cell response after the 2^nd^ vaccination, with 100% and 63% positivity after the 3^rd^ vaccine booster, respectively. Only 25% (1/4) of fingolimod-treated patients had a positive T cell response after the 2^nd^ vaccination and 0% (0/4) after the 3^rd^ vaccination, including patients who were seropositive. In contrast, all ocrelizumab-treated patients (5/5) had a positive T cell response after the 2^nd^ vaccine, despite 58% being seronegative, and 86% (6/7) showed a T cell response after the 3^rd^ vaccine (**Table 3**). One teriflunomide-treated patient did not develop an IFNγ-T cell response post 3^rd^ vaccination, while all of the remaining patients treated with various other DMTs had a positive T cell response.

**Table 3:** Serological and IFNγ-T cell immune response.

Participant	N	Time-point 1: 2-16w post 2^nd^ vaccine		Time-point 2: ~6m post 2^nd^ vaccine		Time-point 3: 1-16w post 3^rd^ vaccine	
		IgG response	T cell response	IgG response	T cell response	IgG response	T cell response
Untreated	1	+	+				
	2					+	+
	3					+	+
	4			+	+		
	5	+	+				
Cladribine	1			+	+		
Dimethyl	1	+	+				
Fumarate	2	+	+				
	3					+	+
Fingolimod	1	+	–			+	–
	2	+	–				
	3	–	+				
	4	–	–				
	5					–	–
	6					–	–
	7					–	–
Ponesimod	1	+	+				
Siponimod	1	+	+				
Glat. Acetate	1	+	+				
IFNβ (Rebif)	1	+	+				
Methotrexte	1	+	+				
Natalizumab	1	+	+				
	2	+	+				
	3	+	+				
	4	+	+				
Ocrelizumab	1	–	+				
	2	+	+				
	3	–	+			–	+
	4	–	+				
	5					–	+
	6					–	+
	7					+	+
	8			–	+		
	9					+	–
	10					+	+
	11					+	+
Ofatumumab	1	+	+				
	2	+	+			+	+
Teriflunomide	1					+	–
Healthy	1	+	+				
	2	+	+				
	3	+	+				
	4	+	+				
	5	+	+				
	6	+	+				
	7					+	+
	8					+	+

+: IgG≥ 50 AU/ml serology, or ≥ 25 pg/ml of Spike-specific IFNγ response. -: IgG<50AU/ml serology, or <25 pg/ml of Spike-specific IFNγ response.

## Discussion

To the best of our knowledge, this is the first comprehensive prospective study on the magnitude and durability of SARS-CoV-2 IgG levels combined with T cell responses in PwMS, treated with various DMTs, for over 6 months from vaccination and following a 3^rd^ vaccine booster. Vaccination is the major available tool to control and fight the pandemic of COVID-19. Several vaccines have been developed worldwide, using different strategies and platforms, including the novel strategy of vaccination with the SARS-CoV-2 spike protein mRNA as in the case of the BNT162b2 (Pfizer/BioNTech,Inc) and the mRNA-1273 (Moderna Tx,Inc) vaccines. These mRNA vaccines appear to be safe and have not been associated with increased incidence of MS disease activation (13–16). In general, the use of DMTs in PwMS has not been found to significantly affect COVID-19 disease course (17), although anti-CD20 therapies have been associated with higher incidence of COVID-19 infection (18) and with a possibly increased risk of severe disease course (19).

An effective, long-term, memory immune response is driven by the adaptive immune system, consisting of both a humoral response mediated by B cells, producing antigen-specific antibodies, and a T cell-mediated response, causing destruction of virus-infected cells and necessary for the development of plasma cells and memory B cells. Thus, measurement of both the humoral and the cell-mediated responses is required to precisely estimate the immune response to SARS-CoV-2 vaccine. However, the relatively easily accessible measurement of anti SARS-CoV-2 antibody levels in the serum is the common and mostly used method to identify individuals who recovered from COVID-19 infection or to confirm a sufficient vaccine response, especially since SARS-CoV-2 neutralizing antibodies were shown to be highly predictive of SARS-CoV-2 immune protection (6). Evidence for positive humoral immune responses to the SARS-CoV-2 vaccine in MS patients is accumulating (9, 10, 12, 20, 21). Our current study provides longitudinal accumulating data regarding the IgG serum levels following >6 months follow-up and also after a 3^rd^ vaccine booster. Our findings show that IgG levels to the SARS-CoV-2 spike protein in MS patients were slightly lower than in healthy individuals, declined by >80% within 6 months from the 2^nd^ vaccination, and were significantly increased following a 3^rd^ vaccine booster. A similar decline in IgG levels at 6 months from vaccination and a beneficial effect of the 3^rd^ vaccine booster was also demonstrated in the healthy cohort. Interestingly, we did not detect a negative correlation between IgG levels after COVID-19 infection and time, suggesting a more robust and long-lasting immune response to the SARS-CoV-2 virus following natural infection vs vaccination. However, the median time between COVID-19-infection and blood collection in our cohort was 27 weeks, thus may not being able to capture the major, initial decline in IgG levels. Antibody levels following COVID-19 infection were shown to be associated with COVID-19 disease severity (a parameter that was not registered in our study), and were reported to start declining after 60 days, but still to be detectable for at least 120 days (22). For the COVID-19-recovered patients in our study, the follow up data were limited, as the majority of these patients received the recommended post–infection vaccination. Interestingly, we found higher IgG levels in vaccinated female than in male patients, not reported in previous reports (20, 23). While we did not detect a similar difference in IgG levels between genders in HC, probably due to the relatively small cohort, both levels of IgGs and of neutralizing antibodies have been shown to be higher in females than in males receiving the BNT162b2 Covid-19 Vaccine, especially in association with age (24), a difference that at least in part is thought to be hormone-mediated (25). The negative correlation between IgG levels and age in vaccinated PwMS or healthy participants presented in our study has also been suggested elsewhere (11, 26), and confirms the potential elevated risk of COVID-19 infection in elderly people.

Our data show, similarly to previous reports, that MS patients treated with most DMTs develop a positive humoral and cell-mediated immune response to the mRNA vaccine, which in general does not differ significantly from that observed in untreated patients or healthy individuals. The reduced humoral response and low frequency of seropositive patients that was observed in PwMS treated with fingolimod and ocrelizumab are in line with several recent studies (7, 8, 10–12, 20, 21, 27–30). In addition, we could show that a similar humoral response and low frequency of seropositive patients is also observed after the 3^rd^ vaccine booster in the patients treated with fingolimod or ocrelizumab, and that in those patients (in contrast to healthy individuals and PwMS in general), the 3^rd^ vaccination did not boost the IgG levels further than the first 2 vaccine doses. One recent study reported similarly that only 1 out of 16 ocrelizumab-treated patients was seropositive after the 3^rd^ BNT162b2 vaccine booster (29). It seems thus, that while some PwMS treated with S1PR-modulators or anti-CD20 therapies do benefit from the additional vaccine dose, an optional vaccine strategy for these patients should be considered. On the other hand, we found that the vast majority of ocrelizumab-treated patients developed a positive anti-spike protein IFNγ-T-cell immune response (both after the 2^nd^ and the 3^rd^ vaccinations) and thus, despite the lack of a sufficient humoral response, they may carry a relative protection against COVID-19 infection or severe disease. Similar results were recently reported by other investigators: Gadani et al. found that 96.9% of ocrelizumab patients developed a T-cell response (30), Tortorella et al. reported a 92% positive T-cell response rate (21) and Brill et al. found a response rate of 89.7% (11). Aposolidis et al. reported positive CD-4 and CD-8 T-cell responses to vaccination in all anti CD-20-treated patients, but these responses were somewhat skewed, with reduced follicular helper T (T_FH_) cell development and elevated CD8 T-cell responses, while Th1 responses were preserved, especially in patients who were seronegative (31). Although these findings of relatively preserved T-cell-mediated anti-COVID-19 responses are encouraging, studies with larger patient cohorts, including follow-up on the risk and outcome of COVID-19 infection are needed to accurately estimate the vaccine-induced protection in anti-CD20- treated patients. Interestingly, we observed both positive humoral and cellular immune responses to the vaccine in patients treated with another anti-CD20 mAB - ofatumumab. We are not aware of published data on the response to vaccination in these patients, however, one study found that COVID-19- recovered ofatumumab-treated, B-cell depleted patients were seronegative for anti SARS-Cov-2 antibodies, but developed a positive cellular response (32), while another study reported a positive anti-SARS-CoV-2 antibody response after COVID-19 in a B-cell depleted ofatumumab-treated patient (33). Confirmation of this finding in a larger group of ofatumumab-treated patients will be of high interest.

Anti-CD20 therapies such as ocrelizumab reduce the number of circulating B cells, thus preventing a sufficient B cell response to antigens and the development of antibodies, an effect which is likely to persist until sufficient B cell reconstitution occurs. It would therefore be of interest to determine the optimal time interval between the infusion and vaccination that would allow for sufficient reconstitution of B cells to enable an effective humoral immune response. This issue has been studied by a few groups but remains debatable; while in some reports there was a significant correlation between time from last treatment to vaccination and the IgG levels (11, 20, 30), others could not confirm this finding (21). In our study, IgG levels correlated only very weakly with the time between infusion and vaccination, both after the 2^nd^ and the 3^rd^ vaccine administration. One study found that 143 days following ocrelizumab infusion is the time-point when IgG starts to increase (20). We found that a time interval of more than 5 months between ocrelizumab infusion and vaccination allows for higher IgG levels than a time interval of <5 months, and a similar difference was also observed in patients vaccinated ≥6 months vs. those vaccinated <6 months after ocrelizumab infusion. Our data also showed that a higher proportion of ocrelizumab-treated patients were seropositive if they were at least 5 months from last infusion at time of vaccine, and in patients with ≥ 6 months time interval, sero-positivity increased to 75% post the 3^rd^ vaccination. Based on our data, and since the humoral response to the vaccine is effective about 14 days after vaccination (34), we recommend that immunization should be timed to the window of 5 months after the last ocrelizumab dose and two weeks before the next dose of ocrelizumab. It was recently suggested that measuring the association between IgG response and B cell reconstitution, rather than the time interval since infusion, is more useful for determining the optimal timing of vaccination administration in ocrelizumab-treated patients (31). A recent study found that the mRNA-1273 vaccine, containing 100 micrograms of the spike protein mRNA, induced higher IgG levels in MS patients compared to the BNT162b2 mRNA vaccine which contains only 30 micrograms of mRNA (20). Based on this finding, the investigators suggested that the mRNA-1273 vaccine would be preferable as vaccination booster in PwMS on anti-CD20 therapy or fingolimod, who did not develop efficient humoral responses following BNT162b2 vaccination.

Our findings indicate that the vast majority of patients treated with fingolimod fail to mount an IFNγ-T-cell immune response. Tortorella et al. similarly found a T-cell response in only 14.3% of fingolimod-treated patients (21). With both reduced or insufficient humoral and cell-mediated immune responses, these patients may be at increased risk of COVID-19 infection and severe disease. Interestingly, it has been suggested that the immunosuppressive effects of fingolimod could be beneficial for prevention of acute respiratory distress syndrome in patients with severe COVID-19, by reducing the cytokine storm (35), and in a recent study fingolimod-treated PwMS were not found to be at increased risk of severe COVID-19 (19). Our data did not confirm a previous report of correlation between IgG response and lymphocyte count in fingolimod-treated patients (20).

Our study has certain limitations. Due to the rapid vaccination program in Israel, the serum samples after both the 2^nd^ and the 3^rd^ vaccines were collected during a variable and rather long time period (2-16 weeks, median ~7 weeks) when patients visited the outpatient clinics, and thus the antibody measurement does not represent the peak of post-vaccination humoral response in every patient. Since there is a natural reduction in IgG levels with time, adjustment for time between vaccination and blood collection was integrated in the statistical analysis. The number of patients treated with other than fingolimod or ocrelizumab S1PR-modulators or anti-CD20 therapies was small, thus limiting any conclusion-making on their effects on the vaccine response. The number of patients assessed for T-cell mediated responses (assessed only in a sub-group of patients) was also low and not sufficiently representative for each DMT, but still our findings add to information emerging from other sites. Data on COVID-19 infection before and during the follow-up period were based only on reports by the participants attending the clinics, and was not intended for interpretation regarding the efficacy of vaccination and the risk of infection in our study population. Since our samples were collected before the outbreak of the latest Omicron variant in Israel, which seems to have the capability to escape immune-surveillance, the impact of the vaccination program on Omicron and future variants is yet to be determined. However, the information on the immune responses of PwMS after the first 3 mRNA vaccines is likely to be relevant for future vaccine development.

The merit of this study is the longitudinal follow-up of IgG levels and cell-mediated responses after 2 vaccinations and the response to a 3^rd^ vaccine booster in PwMS treated with a wide variety of DMTs. While for most DMTs, the humoral and cell-mediated responses appear to be similar to those of untreated PwMS, the finding of sero-negativity in >50% of patients treated with the S1PR-modulator fingolimod and the anti-CD20 therapy ocrelizumab even after a 3 vaccine booster, along with the lack of cell-mediated immune response in the vast majority of fingolimod-treated patients, may suggest that the strategy of boosting the immune system with additional vaccine doses is not effective enough for these patients and that other vaccination strategies should be considered. Such considerations could include optimization of the timing between vaccine administrations, specifically in the context of MS immunotherapies, use of combinational vaccines based on different development platforms and targets, optimization of the vaccine dose and choice of appropriate vaccine in relation to its ability to induce higher antibody levels. In general, efforts should be focused on development of optimal vaccine strategies aiming at improving immunogenicity and long-lasting immunity, tailored for each PwMS under treatment with a specific immunotherapy. Based on the cumulative data until today, updated recommendations about the type and timing of SARS-CoV-2 vaccinations of MS patients are needed.

## Data Availability Statement

The raw data supporting the conclusions of this article will be made available by the authors, without undue reservation.

## Ethics Statement

The studies involving human participants were reviewed and approved by Helsinki Committee, Barzilai Medical Center; Helsinki Committee, Carmel Medical Center; Helsinki Committee, Hadassah Medical Center; Helsinki Committee, Tel Aviv Sourasky Medical Center; Helsinki Committee, Rabin Medical Center;. The patients/participants provided their written informed consent to participate in this study.

## The Israeli Neuroimmunology Study Group on COVID-19 vaccination in Multiple Sclerosis

The Israeli Neuroimmunology Study Group on COVID-19 vaccination in Multiple Sclerosis:

**Lea Glass-Marmor**, Carmel Medical Center; **Anat Volkovitz**, Carmel Medical Center; **Sara Dishon**, Carmel Medical Center; **Zeev Dishon**, Carmel Medical Center; **Netta Kugelman**, Carmel Medical Center; **Zeev Nitsan**, Barzilai Medical Center; **Marwan Alkrenawi**, Barzilai Medical Center; **Nurit Hovel**, Barzilai Medical Center; **Nir Michal**, Barzilai Medical Center; **Vered Loew-Shavit**, Barzilai Medical Center; **Lital Mizrahi**, Barzilai Medical Center; **Marsel Zafrani**, Barzilai Medical Center; **Panayiota Petrou**, Hadassah Medical Center; **Nour Eddine-Yaghmour**, Hadassah Medical Center; **Ariel Ginzberg**, Hadassah Medical Center; **Ibrahim Kassis**, Hadassah Medical Center; **Michelle Halimi**, Hadassah Medical Center; **Keren Regev**, Tel Aviv Sourasky Medical Center; **Hadar Kolb**, Tel Aviv Sourasky Medical Center; **Ifat Vigiser**, Tel Aviv Sourasky Medical Center; **Yoav Piora**, Tel Aviv Sourasky Medical Center; **Irina Komarov**, Tel Aviv Sourasky Medical Center; **Avigail Hindi**, Tel Aviv Sourasky Medical Center; **Adi Wilf-Yarkoni**, Rabin Medical Center; **Itay Lotan**, Rabin Medical Center; **Elia Uri**, Israel Institute for Biological Research; **Shaher Rotem**, Israel Institute for Biological Research; **Hila Cohen**, Israel Institute for Biological Research.

## Author Contributions

AM conceptualized and designed the study, contributed to acquisition and interpretation of the data and revised the manuscript for intellectual content. ES-R designed the study, analyzed and interpreted the data, drafted the manuscript. RM, DK and AK contributed to the design of the study, acquisition and interpretation of the data, and revised the manuscript for intellectual content. MH contributed to acquisition and interpretation of the data and revised the manuscript for intellectual content. EB-H contributed to the acquisition and analysis of the data. The Israeli Neuroimmunology Study Group on COVID-19 vaccination in Multiple Sclerosis contributed to acquisition of the data. All authors contributed to the article and approved the submitted version.

## Funding

This study was coordinated and funded by the Israel Neuroimmunological Society through grants received by Merck, Roche and Novartis companies. The funding sources were not involved in the study design, collection, analysis, interpretation of data, the writing of this article or the decision to submit for publication.

## Conflict of Interest

The authors declare that the research was conducted in the absence of any commercial or financial relationships that could be construed as a potential conflict of interest.

## Publisher’s Note

All claims expressed in this article are solely those of the authors and do not necessarily represent those of their affiliated organizations, or those of the publisher, the editors and the reviewers. Any product that may be evaluated in this article, or claim that may be made by its manufacturer, is not guaranteed or endorsed by the publisher.

## Supplementary Material

Supplementary Figure 1 **(A)** Correlation between IgG after 2^nd^ vaccine and time between Ocrelizumab infusion and 1^st^ vaccine (N=81). **(B)** Correlation between IgG post 3^rd^ vaccine and time between Ocrelizumab infusion and 3^rd^ vaccine (N=40). OCR- ocrelizumab. Dashed horizontal grey line represents minimum seropositive border (log10(50AU/ml).
