# Supplementary figures and images for: Humoral and Cellular Immune Responses to SARS-CoV-2 mRNA Vaccination in Patients with Multiple Sclerosis: An Israeli Multi-Center Experience Following 3 Vaccine Doses
Source: Front Immunol. 2022 Apr 1;13:868915. doi: 10.3389/fimmu.2022.868915 (PMC9012137; doi:10.3389/fimmu.2022.868915)

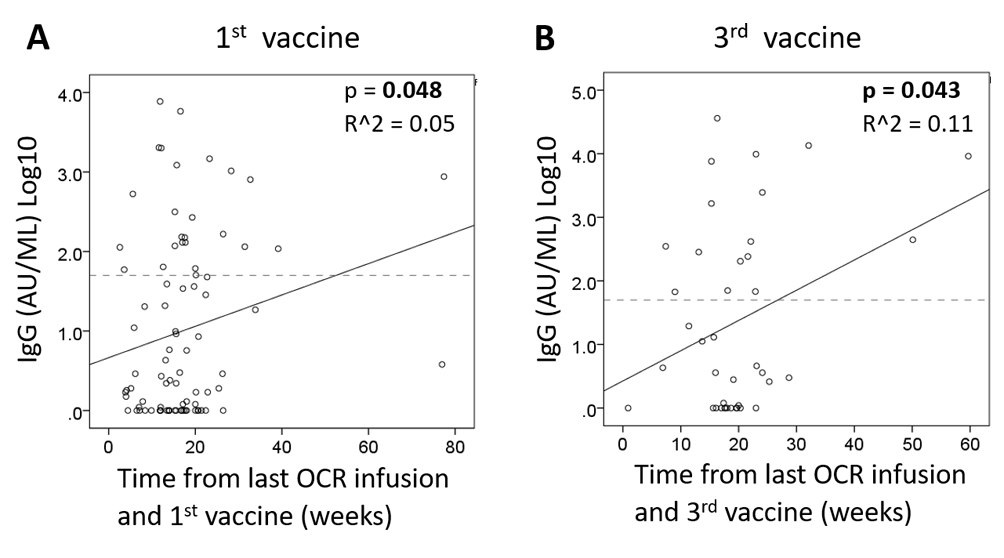

Supplement: Supplementary Figure 1 — (A) Correlation between IgG after 2nd vaccine and time between Ocrelizumab infusion and 1st vaccine (N=81). (B) Correlation between IgG post 3rd vaccine and time between Ocrelizumab infusion and 3rd vaccine (N=40). OCR- ocrelizumab. Dashed horizontal grey line represents minimum seropositive border (log10(50AU/ml). [file Image_1.tif]
