# Supplementary material for: Humoral and Cellular Immune Responses to SARS-CoV-2 mRNA Vaccination in Patients with Multiple Sclerosis: An Israeli Multi-Center Experience Following 3 Vaccine Doses
Source: Front Immunol. 2022 Apr 1;13:868915. doi: 10.3389/fimmu.2022.868915 (PMC9012137; doi:10.3389/fimmu.2022.868915)
Supplement: Supplementary file 2 [file Table_1.docx]

**Supplemental Table 1**

**IgG index following SARS-CoV-2 vaccination or COVID-19 for additional DMTS**

| IgG AU/mL  Mean ± SE  [range]  % IgG positive | Post COVID-19  no vaccine | Time-point 1:  ≤3m  Post 2^nd^ vaccine | Time-point 2:  ~6m  Post 2^nd^ vaccine | Time-point 3:  ≤3m  Post 3^rd^ vaccine |
| --- | --- | --- | --- | --- |
| MS patients | | | | |
| Anti CD-20 mAb  (Rituximab) |  | N=2  43 ± 39  [4 – 82]  50% |  |  |
| Azathioprine |  | N=1  527  100% | N=2  1480 ± 852  [628– 2332]  100% |  |
| Diroximel Fumarate |  |  | N=1  308 100% |  |
| Interferon β (Avonex) | N=1  22699  (100%) | N=8  9376 ± 4517  [1735 – 40000]  100% | N=9  2477 ± 1589  [80 – 14783]  100% | N=5  19450 ± 7163  [2014 – 37181]  100% |
| Interferon β (Betaferon) |  | N=2  1457 ± 683  [775 – 2140]  100% | N=1  733  100% | N=3  9940 ± 4343  [1649 – 16330]  100% |
| Interferon β (Plegridy) |  |  | N=1  157 100% |  |
| Interferon β  (Rebif) |  | N=9  7876 ± 2312  [248 – 23250]  100% | N=4  389 ± 161  [35 – 705]  75% | N=5  17510 ± 6117  [3995 – 40000]  100% |
| S1PR modulator (Ponesimod) |  | N=2  174 ± 12  [162 – 186]  100% |  | N=1  57  100% |

AU- arbitrary units, IgG – immunoglobulin G, m- months, mAb – monoclonal antibody, SE- standard error, S1PR-Sphingosine 1-phosphate receptor.
