# Supplementary material for: Humoral and Cellular Immune Responses to SARS-CoV-2 mRNA Vaccination in Patients with Multiple Sclerosis: An Israeli Multi-Center Experience Following 3 Vaccine Doses
Source: Front Immunol. 2022 Apr 1;13:868915. doi: 10.3389/fimmu.2022.868915 (PMC9012137; doi:10.3389/fimmu.2022.868915)
Supplement: Supplementary file 3 [file Table_2.docx]

| Time interval from OCR to vaccine | 2^nd^ vaccine | | | Time interval from OCR to vaccine | | 3^rd^ vaccine | | | |
| --- | --- | --- | --- | --- | --- | --- | --- | --- | --- |
|  | Mean logIgG | Median logIgG | % sero-positive |  | Mean logIgG | | Median logIgG | % sero-positive |  |
| <1m  N=5  ≥1m  N=81 | 0.897±0.417  1.013± 0.126  *p=0.92, F=0.01* | 0.255  0.462 | 2/5  (40%)  23/81  (28%) | <1m  N=3  ≥1m  N=39 | 0  1.408±0.236  *p=0.37, F=0.825* | | 0  0.663 | 1/1  (0%)  16/39  (41%) |  |
| <2m  N=16  ≥2m  N=70 | 0.615±0.207  1.087±0.139  *p=0.42, F=0.66* | 0.23  0.607 | 3/16  (19%)  22/70  (31%) | <2m  N=3  ≥2m  N=37 | 1.059±0.765  1.398±0.246  *p=0.75, F=0.10* | | 0.634  0.663 | 1/3  (33%)  15/37  (41%) |  |
| <3m N=26  ≥3m N=60 | 0.894±0.241  1.055±0.139  *p=0.49, F=0.49* | 0.241  0.695 | 7/26 (27%)  18/60  (30%) | <3m  N=5  ≥3m N=35 | 1.259±0.44  1.389±0.26  *p=0.89, F=0.03* | | 1.29  0.556 | 2/5  (40%)  14/35  (40%) |  |
| <4m N=52  ≥4m N=34 | 0.939±0.159  1.123±0.187  *p=0.41, F=0.65* | 0.361  0.843 | 13/52  (25%)  12/34  (35%) | <4m N=15  ≥4m N=25 | 1.542±0.385  1.272±0.297  *p=0.57, F=0.34* | | 1.29  0.556 | 6/15  (40%)  10/25  (40%) |  |
| <5m N=70  ≥5m N=16 | 0.889±0.13  1.519±0.29  ***p=0.039****, F=4.43* | 0.342  1.567 | 18/70  (26%)  7/16  (44%) | <5m N=27  ≥5m N=13 | 1.031±0.259  2.082±0.423  ***p=0.036,*** *F=4.76* | | 0.447  2.38 | 8/27  (30%)  8/13  (62%) |  |
| <6m N=76  ≥6m N=10 | 0.909±0.125  1.749±0.351  ***p=0.026****, F=5.19* | 0.342  2.048 | 19/76  (25%)  6/10  (60%) | <6m N=36  ≥6m N=4 | 1.214±0.230  2.804±0.843  ***p=0.042****, F=4.43* | | 0.595  3.305 | 13/36  (36%)  3/4  (75%) |  |

**Supplemental Table 2**

**IgG levels in ocrelizumab-treated patients according to the time interval between last treatment and vaccine.**

Comparison of IgG levels after 2^nd^ or 3^rd^ vaccine between patients with < or ≥ 1,2, 3, 4, 5 or 6 months time intervals between last ocrelizumab infusion and 1^st^ or 3^rd^ vaccination, using a general linear model adjusted for time between vaccination and blood collection. Seropositive border line: Log(50 AU/ml)= 1.699.

IgG- immunoglobulin G, m= months, OCR- ocrelizumab
