# Supplementary material for: Humoral and Cellular Immune Responses to SARS-CoV-2 mRNA Vaccination in Patients with Multiple Sclerosis: An Israeli Multi-Center Experience Following 3 Vaccine Doses
Source: Front Immunol. 2022 Apr 1;13:868915. doi: 10.3389/fimmu.2022.868915 (PMC9012137; doi:10.3389/fimmu.2022.868915)
Supplement: Supplementary file 4 [file Table_3.docx]

|  | Time-point 1  2-16w post 2^nd^ vaccine | | Time-point 2  ~6m post 2^nd^ vaccine | | Time-point 3  1-16w post 3^rd^ vaccine | |
| --- | --- | --- | --- | --- | --- | --- |
| Participant | Mean IgG (AU/ml)  % positive | Mean IFNγ  (pg/ml)  % positive | Mean IgG (AU/ml)  % positive | Mean IFNγ  (pg/ml)  % positive | Mean IgG (AU/ml)  % positive | Mean IFNγ  (pg/ml)  % positive |
| Untreated | 2447 (N=2)  100% | 322  100% | 275 (N=1)  100% | 168  100% | 5577 (N=2)  100% | 208  100% |
| Cladribine |  |  | 296.3 (N=1)  100% | 54  100% |  |  |
| Dimethyl Fumarate | 18369 (N=2)  100% | 6309  100% |  |  | 3243  (N=1)  100% | 52  100% |
| Fingolimod | 94 (N=4)  **50%** | 46  **25%** |  |  | 59.5 (N=4)  **25%** | 0  **0%** |
| Ponesimod | 186 (N=1)  100% | 249  100% |  |  |  |  |
| Siponimod | 301 (N=1)  100% | 35  100% |  |  |  |  |
| Glatiramer Acetate | 3357 (N=1)  100% | 81  100% |  |  |  |  |
| Interferon beta (Rebif) | 1082 (N=1)  100% | 121  100% |  |  |  |  |
| Methotrexate | 1258 (N=1) 100% | 117  100% |  |  |  |  |
| Natalizumab | 19571 (N=4)  100% | 1426  100% |  |  |  |  |
| Ocrelizumab | 368 (N=4)  **25%** | 882  100% | 18.9 (N=1)  **0%** | 758  100% | 357 (N=7)  **57%** | 837  **86%** |
| Ofatumumab | 2989 (N=2)  100% | 170  100% |  |  | 10230.4 (N=1)  100% | 347  100% |
| Teriflunomide |  |  |  |  | 568 (N=1)  100% | 0  **0%** |
| Healthy | 3561.3 (N=6)  100% | 401  100% |  |  | 4429  (N=2)  100% | 87.5  100% |

**Supplemental Table 3 Serological and IFNγ-T cell immune response**
